# Impact of Baizhu, Daqingye, and Hehuanhua extracts on the human gut microbiome

**DOI:** 10.3389/fcimb.2023.1298392

**Published:** 2023-12-07

**Authors:** Johanna M. S. Lemons, Adrienne B. Narrowe, LinShu Liu, Jenni Firrman, Karley K. Mahalak, Pieter Van den Abbeele, Aurélien Baudot, Stef Deyaert, Yanfang Li, Liangli (Lucy) Yu

**Affiliations:** ^1^United States Department of Agriculture, Agricultural Research Service, Eastern Regional Research Center, Dairy and Functional Foods Research Unit, Wyndmoor, PA, United States; ^2^Cryptobiotix, Ghent, Belgium; ^3^Department of Nutrition and Food Science, 0112 Skinner Building University of Maryland, College Park, MD, United States

**Keywords:** polyphenols, intestinal microecology, Hehuanhua, Baizhu, Daqingye, metagenomics, natural products, human health

## Abstract

**Introduction:**

In traditional Chinese medicine, the rhizome of *Atractylodes macrocephala* (Baizhu), the leaves of *Isatis indigotica* (Daqingye), and the flowers of *Albizia julibrissin* (Hehuanhua) have been used to treat gastrointestinal illnesses, epidemics, and mental health issues. Modern researchers are now exploring the underlying mechanisms responsible for their efficacy. Previous studies often focused on the impact of purified chemicals or mixed extracts from these plants on cells in tissue culture or in rodent models.

**Methods:**

As modulation of the human gut microbiome has been linked to host health status both within the gastrointestinal tract and in distant tissues, the effects of lipid-free ethanol extracts of Baizhu, Daqingye, and Hehuanhua on the human adult gut microbiome were assessed using Systemic Intestinal Fermentation Research (SIFR^®^) technology (n=6).

**Results and discussion:**

Baizhu and Daqingye extracts similarly impacted microbial community structure and function, with the extent of effects being more pronounced for Baizhu. These effects included decreases in the Bacteroidetes phylum and increases in health-related *Bifidobacterium* spp. and short chain fatty acids which may contribute to Baizhu’s efficacy against gastrointestinal ailments. The changes upon Hehuanhua treatment were larger and included increases in multiple bacterial species, including *Agathobaculum butyriciproducens*, *Adlercreutzia equolifaciens*, and *Gordonibacter pamelaeae*, known to produce secondary metabolites beneficial to mental health. In addition, many of the changes induced by Hehuanhua correlated with a rise in *Enterobacteriaceae* spp., which may make the tested dose of this herb contraindicated for some individuals. Overall, there is some evidence to suggest that the palliative effect of these herbs may be mediated, in part, by their impact on the gut microbiome, but more research is needed to elucidate the exact mechanisms.

## Introduction

Traditional Chinese medicine (TCM) combines practices like acupuncture, Tai chi, and herbal remedies to maintain human health and well-being (Tang et al., 2008). Foods and herbs are considered through the lens of their medicinal qualities. Modern investigations of traditional Chinese herbs often first identify the phytochemicals present in a substance by extracting these mixed compounds and analyzing them via mass spectrometry, UV, IR, or NMR. Tissue culture or animal studies are then performed using either whole extracts, fractionated extracts, or isolated compounds. These studies have helped to ascribe a molecular mechanism to some of the observed effects and have identified new potential therapeutic uses. Plant-based medicines, like plant-based foods, contain numerous polyphenolic compounds, a well-known class of bioactive molecules, which are the focus of much of this research (Zhang et al., 2022).

Diets rich in polyphenols are protective against cardiovascular, metabolic, and neurodegenerative diseases as well as cancers (Kris-Etherton et al., 2002; Samodien et al., 2019; Franco et al., 2023). These qualities are often attributed to the antioxidant and anti-inflammatory effects of the polyphenols themselves, though the bioavailability of these molecules may be limited due to food matrix interactions and clearance via xenobiotic metabolism (Cardona et al., 2013; Di Lorenzo et al., 2021). Depending on their structure, polyphenols can survive digestion and absorption in the small intestine and arrive in the colon intact where they can be metabolized by the gut microbiota into bioactive secondary metabolites (Cardona et al., 2013; Di Meo et al., 2019). Polyphenols can affect the composition of the gut microbial community by acting as a prebiotic or as an antibacterial agent, especially when glycosylated (Makarewicz et al., 2021). Long-term dietary patterns are a major modulator of the gut microbiome (Fragiadakis et al., 2020), but even short-term changes in diet can impact the microbiome, albeit often transiently (David et al., 2014). Short-term changes may affect the crosstalk between the microbiota and host by changing the composition and function of the microbial community. This crosstalk is often mediated by microbially produced metabolites, some of which have been shown to affect host intestinal barrier function, mood, immunity, fat storage, and disease risk (Krautkramer et al., 2021). This study explored how short-term incubation with polar extracts from three different commonly used Chinese herbs, Baizhu, Hehuanhua, and Daqingye, affected human gut bacteria *ex vivo* and whether any of their beneficial effects could be attributed to interactions with the microbiota.

Baizhu is the name for a common herbal remedy derived from the rhizomes of the indigenous Chinese plant, *Atractylodes macrocephala.* It has most commonly been used to treat gastrointestinal problems like constipation, diarrhea, loss of appetite, and dyspepsia (Zhu et al., 2018). The bioactivity of this herb has largely been attributed to the essential oils, sesquiterpenes, sesquiterpenoids, polysaccharides, and polyacetylenes present in this herb (Zhu et al., 2018; Gu et al., 2019; Zhu et al., 2021; Lv et al., 2022). These chemical components of Baizhu are thought to be responsible for the herbs’ ability to both promote and inhibit intestinal peristalsis, increase gastric mucosal healing and other beneficial effects via varied mechanisms (Yang et al., 2021). Daqingye is the name for the leaves of the *Isatis indigotica* plant which is used both medicinally and as a dye. Daqingye is used to treat influenza and other viral diseases causing sore throat, fever, and flushed skin (Chen et al., 2021). These leaves contain alkaloids, like tryptanthrin, indirubin, and indigotin, with demonstrated antiviral and antibacterial activity, as well as organic acids, flavonoids, and lignans (Chen et al., 2021). Among the organic acids is salicylic acid, the major metabolite of aspirin and known anti-inflammatory compound that might account for Daqingye’s efficacy as a pain and fever reducer. Hehuanhua is the name for the flowers of *Albizia julibrissin* and is known for its sedative effect. It is used in the treatment of mood disorders as well as bruises, fractures, stroke, and poor vision (Lu et al., 2023). The bioactive phytochemicals present in Hehuanhua include triterpenoids, flavonoids, phenols, and alkaloids (Lu et al., 2023). The flavonoids, in particular, are associated with many different pharmacological activities which may influence mental health/mood including anti-inflammatory, antidepressant, anxiolytic, as well as others like anti-obesity, anti-neoplastic and anti-osteoporosis (Huang et al., 2023; Lu et al., 2023).

While these three herbs are prescribed for different conditions, each of them can be ingested orally and therefore encounter the gut microbiota during the digestive process. Extracts from all three herbs were used to investigate the impact on the structure and function of the gut microbiota of six individual donors using the high throughput *ex vivo* SIFR^®^ (Systemic Intestinal Fermentation Research) technology, which has been shown to accurately preserve *in vivo*-derived microbiota throughout the entire duration of the experiment (Van den Abbeele et al., 2023c). Along with shotgun metagenomic sequencing, each culture was analyzed for total cell counts, pH changes, gas production, and short chain fatty acid (SCFA) production. This study has helped generate new hypotheses on how the diverse chemicals within each herb may interact with the gut microbiome to exert their beneficial effects.

## Materials and methods

### Production of herbal extracts

Baizhu, Daqingye, and Hehuanhua were purchased from a TCM retail store as dried preparations (Rockville, MD). The herbs were ground into a particle size <40 mesh with a Micro-Mill^®^ Grinder (Bel-Art Products, Pequannock, NJ). They were then defatted using a solid-liquid extraction method using hexane, at a sample-to-solvent ratio of 1:5 (w/v) in an Erlenmeyer flask for 2 hours. The mixture was then separated by gravity filtration and was left in a fume hood at room temperature overnight to ensure complete evaporation of residual hexane. The dried, defatted sample was extracted with 95% ethanol by Soxhlet extraction (Choe et al., 2021). The solvent was finally removed by rotatory evaporation. The dried extracts were re-grounded to a fine powder and stored at -20°C until used in the bacterial culture experiments.

### *Ex vivo* culturing experiments

Fecal samples were collected from six adult donors ages 25-40 years old with a BMI between 18 and 30 who were not pregnant or lactating, according to the IRB protocol approved by the Ethics Committee of the University Hospital Ghent, Belgium (BC-09977). Donors were non-smokers, drank less than three servings of alcohol/day, had no gastrointestinal disorders or cancer, were not on any medications to treat psychological disorders or allergies, and had no anti-/pre-/probiotics for at least three months before their donations. Three donors were male and three were female.

**Figure 1:**
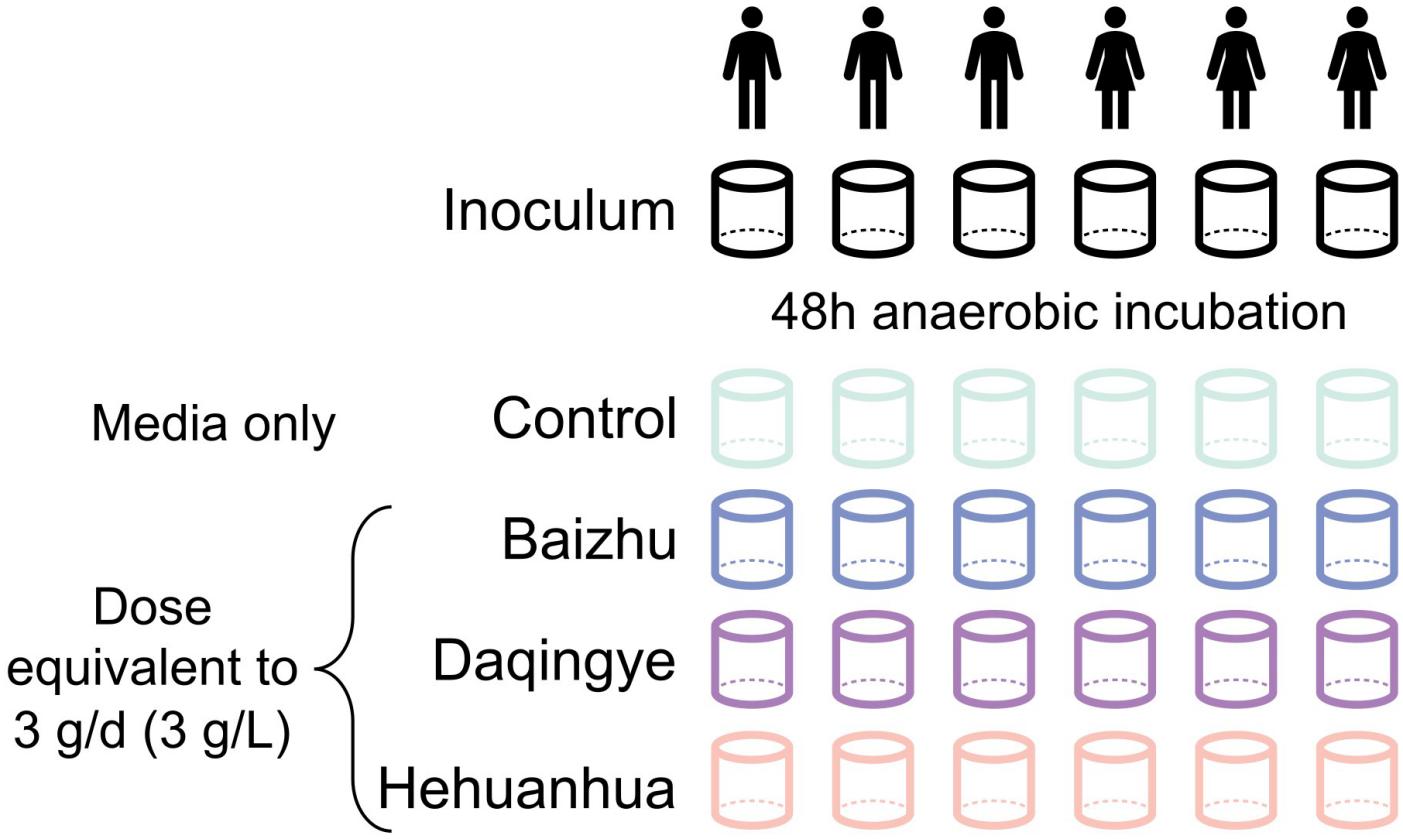
Diagram of experimental design.

Gut microbiota testing was performed using *ex vivo* SIFR^®^ technology as described previously (Van den Abbeele et al., 2023c). Briefly, anaerobic bioreactors containing anaerobically prepared nutritional media were inoculated with fecal slurry. For each donor, one bioreactor contained nutritional media only (Control) and three others contained nutritional media supplemented with 3 g/L of one of three herbal extracts (Baizhu, Daqingye, and Hehuanhua) to simulate a human dose of 3 g/day (**Figure 1**). The background medium used across all incubations was medium M0003 (Cryptobiotix, Ghent, Belgium). Samples were harvested at the time of inoculation (Inoculum) and 48 hours post-inoculation. The environmental pH and gas production were measured during the experiment and harvested samples were used for flow cytometry, shotgun sequencing, and targeted metabolomics.

### Bacterial cell counts

For total bacterial cell count analysis, samples were diluted in anaerobic phosphate-buffered saline, stained with 1 µM SYTO 16, and counted using a BD FACS Verse flow cytometer (BD, Erembodegem, Belgium) (Van den Abbeele et al., 2023a). Data were analyzed using FlowJo, version 10.8.1.

### Environmental pH and gas measurement and SCFA quantification

The environmental pH for each anaerobic culture tube was determined using a Senseline pH meter F410 (ProSense, Oosterhout, The Netherlands), and gas production was measured as the pressure in the headspace of each vessel at the beginning and end of the experiment. SCFAs were extracted using diethyl ether and a 1 µL injection volume was used to detect individual SCFAs with a GC-2014 gas chromatography (Shimadzu) instrument as described previously (Van den Abbeele et al., 2023b). The targeted method quantified acetate, propionate, butyrate, and valerate, as well as branched chain fatty acids (BCFAs), isobutyrate, isovalerate, and isocaproate. Reported total SCFA and BCFA amounts were calculated by summing the respective fatty acids.

### DNA extraction, library preparation, and sequencing

DNA extraction, shotgun metagenomic library preparation, and sequencing were conducted by CosmosID (Germantown, MD.) DNA was isolated using the QIAGEN DNeasy PowerSoil Pro Kit, according to the manufacturer’s protocol. Extracted DNA samples were quantified using a Qubit 4 fluorometer and Qubit™ dsDNA HS Assay Kit (Thermofisher Scientific). DNA sequencing libraries were prepared using the Nextera XT DNA Library Preparation Kit (Illumina) and IDT Unique Dual Indexes with a total DNA input of 1 ng. Genomic DNA was fragmented using a proportional amount of Illumina Nextera XT fragmentation enzyme. Unique dual indexes were added to each sample followed by 12 cycles of PCR to construct libraries. DNA libraries were purified using AMpure magnetic Beads (Beckman Coulter) and eluted in QIAGEN EB buffer. DNA libraries were quantified using Qubit 4 fluorometer and Qubit™ dsDNA HS Assay Kit. Libraries were then sequenced on an Illumina HiSeq X platform 2x150bp to a target depth of ~3M read pairs per sample.

### Read-based taxonomic and functional profiling

Raw reads were preprocessed by adapter removal and quality trimming using BBDuk v.38.79 (Bushnell, 2015) with parameters: (k=31, hdist=1, ftm=5; qtrim=r, trimq=10). Reads were additionally filtered using BBDuk to remove reads mapping to the human genome. Trimmed, filtered reads were used as input for MetaPhlAn4 v. 4.0.6 (Blanco-Miguez et al., 2023), with the mpa_vOct22_CHOCOPhlAnSGB_202212 database to perform read-based taxonomic assignment and estimation of relative abundance. Read-based functional profiles were generated using HUMAnN v.3.6 (Beghini et al., 2021) and normalized to CPM. In addition to gene family and reaction level profiling, the profiles were classified as KEGG orthologs, EC numbers, MetaCyc pathways, and GO terms.

### Taxon and pathway association testing

MaAsLin2 (Microbiome Multivariable Associations with Linear Models) was used to discover specific and significant associations of microbial taxa and functional pathways with each herb (Mallick et al., 2021). For the taxonomic data, log-transformed data (relative abundance data for taxon, and CPM normalized for functional data) was used for all 48-hour samples specifying ‘donor’ as random effects and product as fixed effect specifying ‘Control’ as the reference level according to the per-feature model: feature ~ (intercept) + herb + (1 | donor) where feature is either taxon or pathway depending on the dataset. Multiple testing correction was performed using the Benjamini-Hochberg method with the method’s default FDR threshold of 0.25.

### Other analyses and visualizations

Statistical analyses were conducted using R/RStudio (v.4.1.3) using the packages: tidyverse (v.1.3.1) (Wickham, 2009), vegan (v.2.6-2) (Oksanen et al., 2022), ape (v.5.6-2) (Paradis and Schliep, 2019). Heatmaps of pathways and taxa were created using GraphPad Prism 10 (GraphPad Software, San Diego, CA).

### Data availability

Raw metagenomic sequencing data are available in the NCBI Sequence Read Archive associated with BioProject PRJNA961974.

## Results

**Figure 2:**
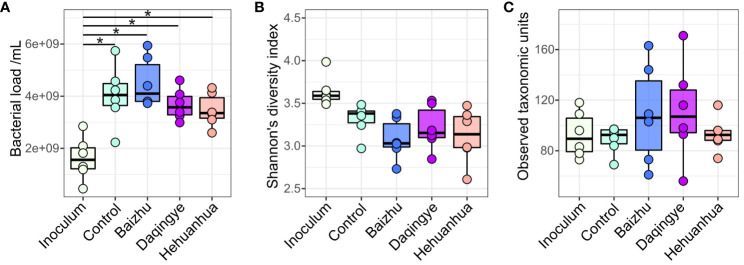
Community structure in terms of **(A)** bacterial load, **(B)** Shannon’s diversity, and **(C)** richness. There were no statistically significant differences between treatments by ANOVA with Tukey’s HSD *post hoc* test, but there was a significant increase in cell number for all treatments compared to the inoculum * indicates adjusted p-value < 0.01.

Fecal samples from six donors were incubated in media alone or with additional administration of 3 g/L of herbal extract for 48 hours according to the diagram in **Figure 1**. Flow cytometry was used to measure total bacterial abundance which showed a significant increase in bacterial load with all treatments compared to inoculum, as would be expected during proliferative growth (**Figure 2A**). Despite a trend toward more bacterial growth in the presence of Baizhu and less in the presence of Hehuanhua, there was no significant difference between cell counts in the herbal treatments and the Control (**Figure 2A**). MetaPhlAn4 was used to estimate community composition based on metagenomic sequencing data which was then used to calculate Shannon’s diversity and numbers of observed taxa (richness). Overall bacterial diversity remained similar across Inoculum and all treatment groups (**Figure 2B**). There was also no change in taxonomic richness observed between the Inoculum, Control, or herbal groups (**Figure 2C**). This indicated the number of identified taxa did not change throughout the study since the numbers in the starting material were similar to those following a 48-hour incubation, which is consistent with previous findings for the *ex vivo* SIFR^®^ platform (Van den Abbeele et al., 2023c).

**Figure 3:**
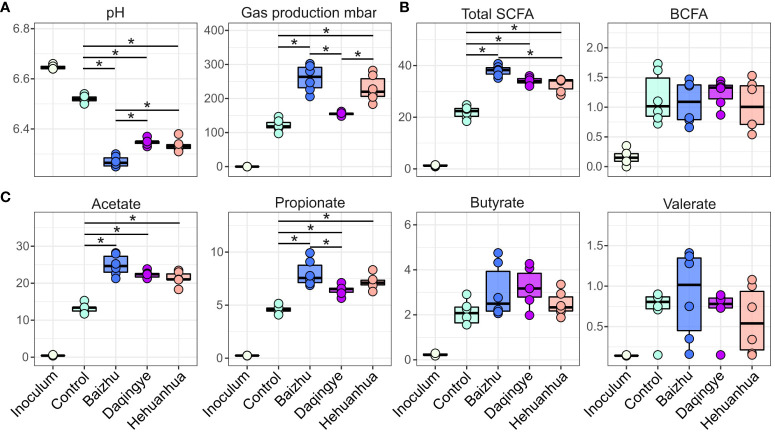
Functional output for the control and treatment groups. Statistical significance was determined by ANOVA for all treatments at 48 hours with Tukey’s HSD post-hoc test; * indicates adjusted p-value < 0.01. **(A)** pH and gas production, **(B)** levels of total SCFAs and BCFAs in mM, and **(C)** levels of individual SCFAs.

pH, gas production, and fatty acid production were used to assess the fermentation activity of the bacterial communities under all treatment conditions (**Figure 3**). For many of the parameters, there were significant changes following incubation (data not shown). Interestingly, all three herbs induced a significant drop in pH compared to the Control, while only Baizhu and Hehuanhua caused a significant rise in gas production (**Figure 3A**). The drop in pH for all herbal treatments corresponds to a rise in SCFAs and not BCFAs for these treatments compared to Control (**Figure 3B**). The rise in SCFAs is predominately driven by increased abundance of acetate and propionate (**Figure 3C**). Significant differences between pH, gas production, and total SCFAs between different herbal treatment groups were also observed.

**Figure 4:**
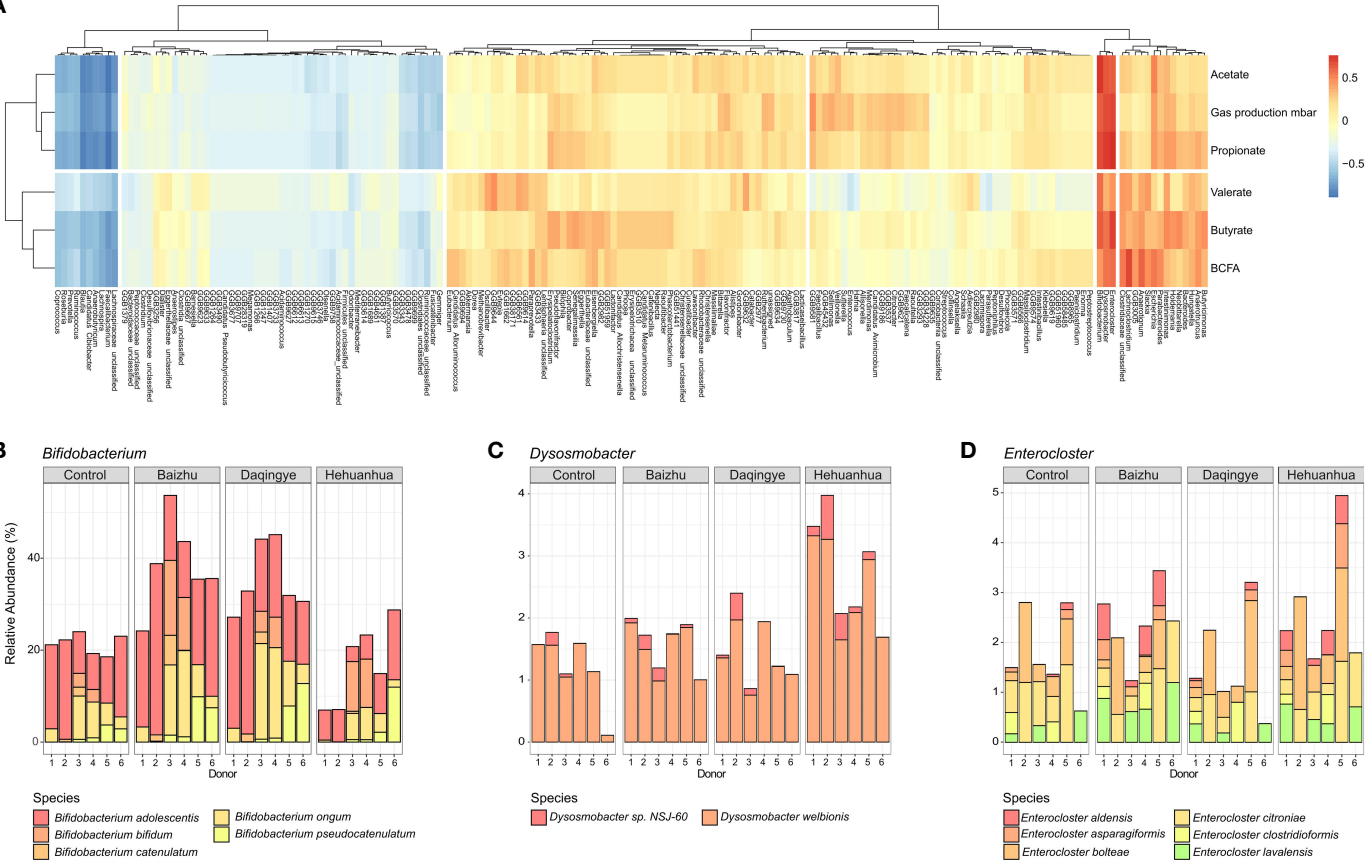
**(A)** Heatmap showing correlations between bacterial taxa and fermentation parameters for all three herbal treatments. The strongest positive correlation between all fermentation parameters and bacterial taxa was for **(B)** *Bifidobacterium* **(C)** *Dysosmobacter* and **(D)** *Enterocloster.* Plots are shown depicting the relative species-level abundances of these taxa.

Across all treatments, the bacteria with the strongest positive correlation with the observed increases in gas and individual SCFA production were those of the *Bifidobacterium, Enterocloster*, and *Dysosmobacter* genera (**Figure 4A**). These three taxa cluster together within the heatmap. Looking more closely at the treatment-specific response, Baizhu and Daqingye both upregulate *Bifidobacterium* spp. (**Figure 4B**), which are well-known saccharolytic bacteria and producers of acetate (Riviere et al., 2016). *Bifidobacterium* spp. are important cross-feeders in the gut supplying acetate and lactate which can be metabolized by other gut bacteria to butyrate, which shows a slight increase with these treatments (**Figure 3C**). Baizhu induces a slight increase in *Enterocloster* spp. which can produce acetate, though there is some debate about whether they also produce butyrate (**Figure 4D**) (Mohan et al., 2006; Warren et al., 2006; Domingo et al., 2009; Haas and Blanchard, 2020). Hehuanhua, on the other hand, decreases *Bifidobacterium* and increases both *Enterocloster* and *Dysosmobacter* (**Figures 4B–D**), known butyrate producers (Le Roy et al., 2020).

**Figure 5:**
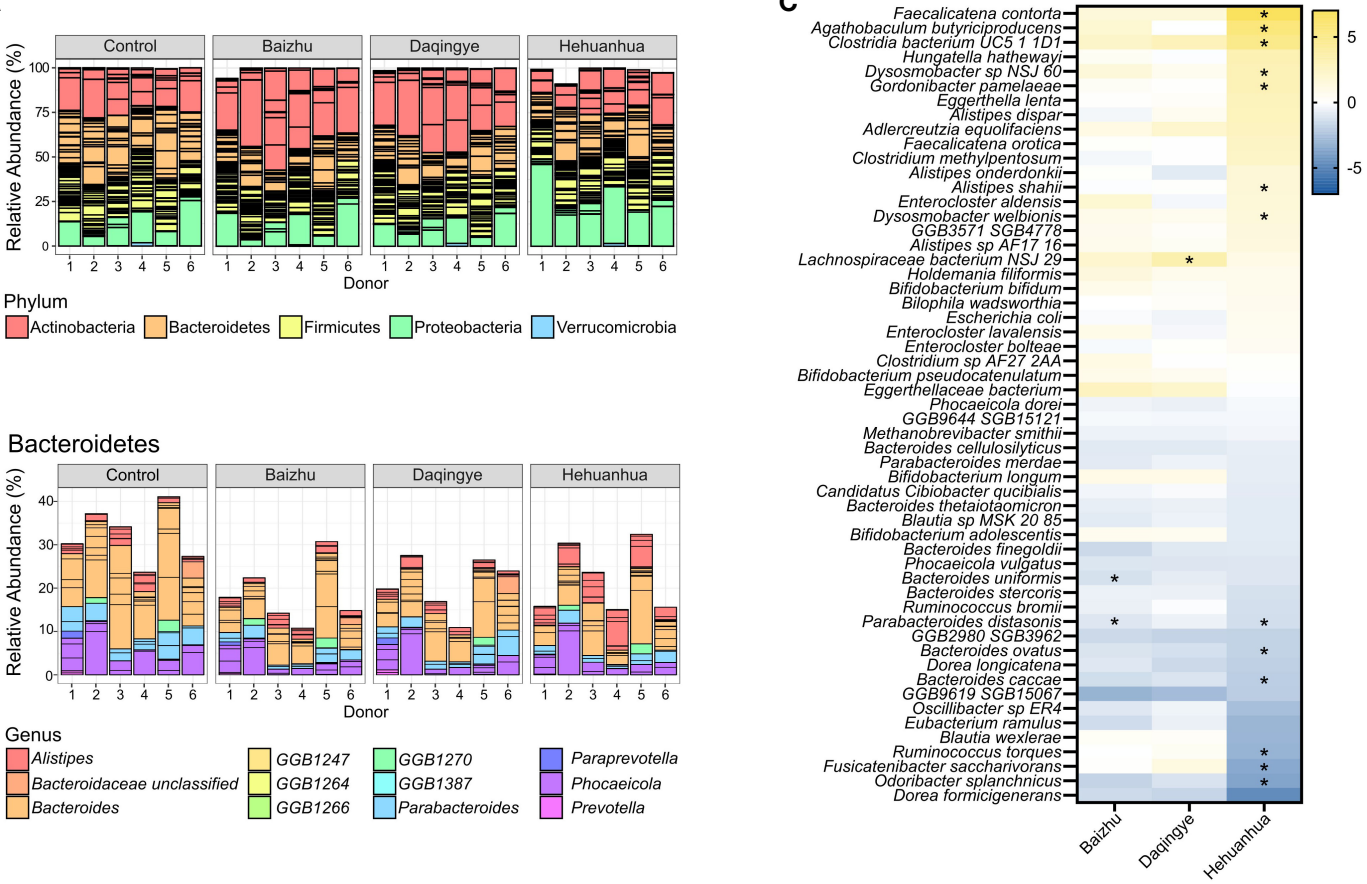
**(A)** Relative abundance of different phyla in the control and herbal treatment groups. **(B)** Bacteroidetes taxonomic changes with treatment broken down by genera present at ≥ 1% relative abundance. **(C)** Heatmap of all species that changed upon treatment compared to NSC with a q-value of ≤ 0.4 in at least one condition, * indicates q ≤ 0.1.

The phylogenetic abundance of the bacterial communities for each donor under each treatment condition was characterized using metagenomic sequencing data (**Figure 5A**). Bacteroidetes abundance was significantly decreased upon incubation with Baizhu extracts compared to Control (p=0.0396) and trended downward with Daqingye and Hehuanhua (p=0.121 and 0.199, respectively). There were increases in Actinobacteria upon incubation with Baizhu and Daqingye (p = 0.221 and 0.322, respectively) and in Proteobacteria with Hehuanhua (p=0.387), but none of these differences reached statistical significance. Within the Bacteroidetes phylum, all three herbs induced significant decreases in members of the *Bacteroides* and *Parabacteroides* genera while Hehuanhua also induced a rise in multiple members of the *Alistipes* genus across donors, which is known to be resistant to toxic compounds (**Figures 5B**, **C**). Some of these results are reflected in the list of bacteria whose abundance changed the most with each treatment condition (**Figure 5C**).

**Figure 6:**
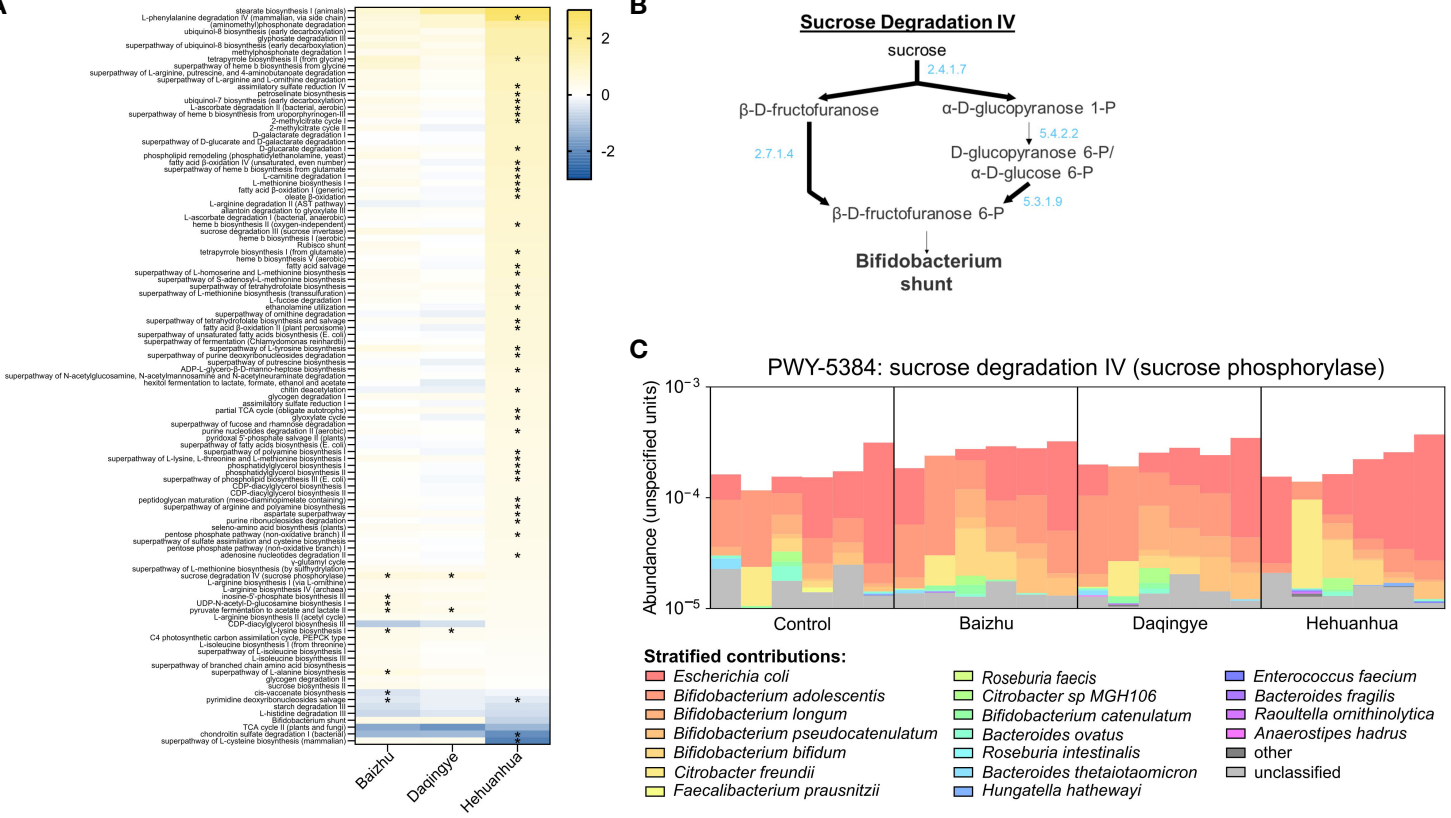
**(A)** All MetaCyc pathways with a q ≤ 0.1 that exhibit a difference compared to NSC for at least one treatment (* indicates q ≤ 0.05). **(B)** Sucrose degradation pathway IV with bold lines indicating genes that are highly abundant in Baizhu and Daqingye treatment groups. **(C)** Shows the relative contribution of different bacterial species to the genes within the sucrose degradation IV pathway.

Humann3 was used to perform read-based functional profiling of the metagenomes and annotation of these profiles using KEGG orthology and MetaCyc pathway terms, converting estimated functional abundances to counts per million. These profiles were used with MaAsLin2 to identify genes and pathways that differed significantly between the herbal treatment groups and the Control to evaluate the relationship between changes in community structure and functional capacity. The changes in gene abundance when comparing the Baizhu and Daqingye treatment groups to the Control were small, in number and magnitude, when compared to those for Hehuanhua treatment (**Supplemental Data**). **Figure 6A** shows all the Metacyc pathways that changed compared to Control for at least one of the herbal treatments with a q-value of ≤ 0.1. For a pathway to be considered changed, multiple genes within that pathway must change. Among the pathways whose genes were more abundant in the presence of Baizhu and Daqingye are many related to active cellular proliferation like sugar metabolism and amino acid, nucleotide, and peptidoglycan biosynthesis (**Figure 6A**). The most significant change for both treatments was in the abundance of genes associated with PWY-5384, the sucrose degradation IV (sucrose phosphorylase) pathway (~0.594 log2fold increase, q = 0.0129 for Baizhu and ~0.555 log2fold increase, q = 0.0129 for Daqingye). **Figure 6B** provides a representation indicting the exact genes within this pathway which were observed to be more abundant after treatment with either Baizhu or Daqingye. PWY-5384 is an important fermentative pathway for *Bifidobacterium* which precedes the *Bifidobacterium* shunt, by which acetate and lactate are produced. **Figure 6C** shows that *Bifidobacterium adolescentis*, *Bifidobacterium longum*, *Bifidobacterium pseudocatenulatum*, and *Bifidobacterium bifidum* are the species largely responsible for the increased abundance of genes in this pathway for Baizhu and Daqingye compared to Control whereas *Escherichia coli* contributes more to this pathway with Hehuanhua treatment. The upregulation of this and other similar pathways indicate that communities treated with Baizhu and Daqingye have a slightly increased genetic potential to perform fermentative metabolism compared to Controls, which agrees with the observed changes seen in fermentative outputs (**Figure 3**).

Of these three herbal extracts, Hehuanhua caused the most abundant and significant changes in the bacterial community in terms of both structure and function (**Figures 5C**, **6A**). 85 different KOs were significantly (q < 0.05) changed with Hehuanhua treatment compared to control. Among these are genes like K03182 4-hydroxy-3-polyprenylbenzoate decarboxylase (~1.86 log2fold increase, q = 0.0107), K10831 taurine transport system ATP binding protein (~1.75 log2fold increase, q = 0.0175), K00390 phosphoadenosine phosphosulfate reductase (~2.15 log2fold increase, q = 0.0271), and K01071 medium-chain acyl-[acyl-carrier-protein] hydrolase (~2.56 log2fold increase, q = 0.0272) (**Supplemental Data**). K03182 is part of the ubiquinol synthesis pathway from chorismate, a chemical that also serves as a precursor to other compounds including tetrahydrofolate, enterobactin, and menaquinol. Increased genetic potential for the synthesis of each of these is also suggested by the genes and pathways whose abundance increases with Hehuanhua treatment. K10831 and K00390 are both involved in sulfur metabolism and K01071 is part of fatty acid biosynthesis.

While the increased abundance of these genes within the community does not necessarily mean increased expression, given the gene sets that are observed, the Hehuanhua-treated bacterial community does have a higher genetic potential to engage in porphyrin biosynthesis (eg. PWY-5918, superpathway of heme b biosynthesis from glutamate; ~0.917 log2fold increase, q = 0.0279), tetrahydrofolate biosynthesis and turnover (eg. FOLSYN-PWY superpathway of tetrahydrofolate biosynthesis and salvage; ~0.683 log2fold increase, q = 0.0390), sulfur assimilation (eg. PWY1ZNC-1 assimilatory sulfate reduction IV; ~1.17 log2fold increase, q = 0.0390) and anaerobic respiratory metabolism (eg. CARNMET-PWY L-carnitine degradation I; ~0.907 log2fold increase, q = 0.0390) (**Figure 6A**; **Supplemental Data**). The bacterial species contributing to the abundance of many of these genes and corresponding pathways are various species of *Escherichia*, *Klebsiella*, and *Citrobacter* in the *Enterobacteriaceae* family (**Supplemental Figure 1**). Surprisingly the species whose relative abundances change the most, *Faecalicatena contorta* (~6.71 log2fold increase, q = 0.00977), *Agathobaculum butyriciproducens* (~5.51 log2fold increase, q = 0.0659)*, Clostridia bacterium UC5-1-1D1* (~4.87 log2fold increase, q = 0.0662)*, Fusicatenibacter saccharivorans* (~3.33 log2fold decrease, q = 0.0982), and *Odoribacter splanchnicus* (~3.51 log2fold increase, q = 0.0982) among others (**Figure 5C**; **Supplemental Data**), were not species within the *Enterobacteriaceae* family. This suggests functional redundancies among the taxa whose abundances did change considerably. Among the *Enterobacteriaceae*, the species whose abundance change was most significant was *Escherichia coli* (~0.642 log2fold increase, q = 0.318) (**Figure 5C**; **Supplemental Data**). However, the abundance of the Proteobacteria phyla, to which the *Enterobacteriaceae* belong, did increase slightly with Hehuanhua treatment (**Figure 5A**).

## Discussion

While some studies have been performed to elucidate the molecular mechanisms by which the chemical compounds found in herbal remedies affect mammalian physiology, much less has been done to investigate how these medicines affect the host via the microbiome. The study discussed here aimed to investigate the impact of extracts from three herbs used in TCM on the human gut microbiome *ex vivo*. Fermentation outputs including gas production and SCFA production were monitored and shotgun metagenomic sequencing was performed to assess the effect on the bacterial communities in terms of structure and functional potential. Each of these herbs is used to treat different ailments, but all can be consumed orally indicating a potential for them to interact with the gut microbiota within the gastrointestinal tract. Other researchers have performed experiments investigating the effect of Shenling Baizhu powder, a formulation of ten herbs including Baizhu, on the gut microbiome of rats, but studies have not been performed to assess the effect of these herbs individually on the human microbiome (Zhang et al., 2018).

Baizhu induced a few small but significant changes in the microbial community such as increased abundance of *Bifidobacterium* spp. (**Figure 4B**). The increase in *Bifidobacterium* was also previously seen in rats treated with Shenling Baizhu powder (Zhang et al., 2018). This rise in *Bifidobacterium* partially drives the observed rise in the Actinobacteria phylum (**Figure 5A**). On their own, *Bifidobacteria* have been used as probiotics to treat diarrhea, improve colon regularity, reduce symptoms of inflammatory bowel disease, and even prevent necrotizing enterocolitis in neonates (O'Callaghan and van Sinderen, 2016). In some ways, the rise in *Bifidobacterium* may functionally compensate for the observed loss of *Bacteroidetes* spp. since both are capable of glycan degradation. *Bifidobacterium* uniquely metabolizes hexose sugars through a pathway known as the “*Bifidobacterium* shunt” which is known to produce acetate and lactate (O'Callaghan and van Sinderen, 2016) and would contribute to the high levels of acetate and lower pH during Baizhu treatment (**Figures 3A, C**). Lactate produced by these organisms can then cross-feed other organisms that produce propionate which also accumulated during incubation with Baizhu (**Figure 3C**) (Reichardt et al., 2014). Metabolically upstream of the Bifidobacterium shunt is the sucrose degradation IV pathway (**Figure 6B**), which is the pathway most affected by Baizhu treatment (**Figure 6A**). Many health benefits are associated with the production of SCFAs, Baizhu consumption may increase the relative abundance of *Bifidobacteria* leading to significant increases in SCFAs which bolster gastrointestinal health (Morrison and Preston, 2016).

In addition to causing a rise in *Bifidobacterium* spp. that may heal the gastrointestinal tract, Baizhu also caused a decrease in some species of Bacteroidetes which have been specifically linked to gastrointestinal harm. Although Bacteroidetes often constitute a major proportion of a healthy microbial community in the gut, their role in health and disease is mixed (Johnson et al., 2017; Gibiino et al., 2018). This phylum is genetically diverse but shares a prowess for polysaccharide utilization through their expression of a high number of carbohydrate-active enzymes. Bacteroidetes can encode enzymes capable of degrading glycans from both plant and host mucosal origin, making them highly metabolically flexible (Johnson et al., 2017). An overabundance of mucus degraders like *Bacteroides caccae* can result in a thinner protective barrier in the host and increased susceptibility to bacterial pathogenesis (Desai et al., 2016). Additionally, increased abundance of *Bacteroides uniformis*, *Bacteroides vulgatus* (now *Phocaeicola vulgatus*) (Garcia-Lopez et al., 2019), *Prevotella falsenii*, and *Parabacteroides distasonis* have all been implicated in the development of inflammatory bowel disease and colitis while *Bacteroides fragilis* has been associated with colorectal carcinoma (Gibiino et al., 2018). Interestingly, the most significant drops in species level abundance in response to Baizhu treatment were for many of these same species; *Parabacteroides distasonis* (**Figure 5C**) followed by *Bacteroides uniformis*, *Parabacteroides merdae*, *Phocaeicola vulgatus* and *Bacteroides caccae* (**Supplemental Data**). The predominant mucin-associated oligosaccharides in the gastrointestinal tract are sialic acid and fucose (Robbe et al., 2003). Genes encoding alpha-L-fucosidase, the presence of which is specifically associated with the *Bacteroides* and *Parabacteroides*, are also less abundant in the collective genome of the Baizhu-treated community (**Supplemental Data**). A drop in these species and other potentially pathogenic or mucus-degrading *Bacteroidetes* may contribute to a thicker mucus layer and greater protection against intestinal inflammation and resulting diarrhea, which is in line with Baizhu’s efficacy against gastrointestinal problems (Zhu et al., 2018).

Daqingye, as mentioned before, is commonly used to treat cold-like symptoms including sore throat, fever, and flushed skin (Chen et al., 2021). Some research has attempted to determine which of the many chemical compounds in this herb are effectors of its beneficial effects. Reports indicate that the three major compounds found within the leaf of *Isatis indigotica*, indigotin, indirubin, and tryptanthrin, all have anti-viral activity (Mak et al., 2004; Tsai et al., 2020). The antipyretic and analgesic attributes of this herb may be the result of the salicylic acid it reportedly contains, which was first identified as the active substance in willow bark from which aspirin was derived (Plaisance and Mackowiak, 2000). Overall, although some of the same changes in the microbial community were observed upon treatment with Daqingye as with Baizhu, the magnitude was generally less and does not explain any reported medicinal qualities of the herb. Given this, it is not possible to determine whether the microbiota mediates any of the specific effects for which Daqingye is often prescribed.

Of the three herbs studied, Hehuanhua induced the most changes to the gut microbial community. Some of the observed changes suggest that the microbiota could be a mediating factor in the palliative effects of Hehuanhua. For instance, genes involved in tetrapyrrole biosynthesis pathways are increased with Hehuanhua treatment, which can be used to synthesize heme and Vitamin B12 (**Figure 6A**). Levels of Vitamin B12 along with tetrahydrofolate, whose pathways are also more prevalent in the microbiome of Hehuanhua-treated communities, are inversely correlated with depression, especially in women (Coppen and Bolander-Gouaille, 2005). Other plant-derived mixtures have also been shown to increase Vitamin B12 production in the gut microbiota (Sun et al., 2023). The link between these co-factors and mental health is likely via the role they play in one-carbon metabolism (SAM cycle) which is important for the production of serotonin and other neurotransmitters (Seppala et al., 2013). There is evidence that other components necessary for active one-carbon metabolism are also present including the increased abundance of genes involved in methionine, cysteine, and glutathione metabolism suggesting that this pathway is also important for the bacterial community (**Figure 6A**; **Supplemental Data**). Glutathione, which is also important for redox balance and detoxification, is depleted in neuro-relevant conditions like autism, Parkinson’s, and Alzheimer’s (Aoyama, 2021; Bjorklund et al., 2021). Interventions that increase the bacterial production of Vitamin B12, folate, glutathione, etc. may be beneficial for the mental health of the host (Coppen and Bolander-Gouaille, 2005).

While there are many bioactive compounds in Hehuan, not all of them are linked to the mental health benefits of the herb (Lu et al., 2023). A few compounds (i.e., quercetin and its glycosides, sulfuretin, (−)-syringaresnol-4-O-β-d-apiofuranosyl-(1→2)-β-d-glucopyranoside, and Julibroside C1) have been specifically linked to the anti-insomnia, anti-depressive, anti-anxiety, and neuroprotective activity with which Hehuanhua is traditionally associated (Chen et al., 2022; Sun et al., 2022; Lu et al., 2023). The neuroprotective effects of sulfuretin in Alzheimer’s disease are thought to occur through activation of nuclear factor erythroid 2-related factor 2 (Nrf2)-dependent heme oxygenase-1 (Ho-1) expression via the PI3K/Akt signaling pathway (Kwon et al., 2015). Interestingly, one species whose abundance increased the most with Hehuanhua treatment was *Agathobaculum butyriciproducens* (**Figure 5C**) and it has also been shown to be neuroprotective in Alzheimer’s and Parkinson’s disease models via Nrf2-induced expression of Ho-1 (Go et al., 2021; Lee et al., 2022). Among the species whose abundance is greatly increased with Hehuanhua treatment are known producers of butyrate, indoles, urolithins, and equol (**Figure 5C**) (Toh et al., 2013; Selma et al., 2014; Ahn et al., 2016; Sakamoto et al., 2017); all compounds that have been associated with neuroprotective or anti-anxiety/depressive effects (Subedi et al., 2017; Bayazid et al., 2021; D'Amico et al., 2021; Vadaq et al., 2022). So, while the literature suggests that the chemical components of Hehuanhua recapitulate the mood-elevating properties of this herb, the data presented here suggests that microbiome changes after Hehuanhua treatment may also contribute.

Finally, while Hehuanhua has well-documented health benefits, it was observed that incubation with this compound also increased the abundance of Proteobacteria (**Figure 5A**), which also showed up as an increase in the abundance of multiple genes/pathways associated with *Enterobacteriaceae* (**Supplemental Figure 1**). Proteobacteria is the phylum most associated with gut dysbiosis, which may make Hehuanhua use, at least at the tested dose, contraindicated for some individuals (Shin et al., 2015). The fact that *Enterobacteriaceae*-associated genes change the most, even though individual *Enterobacteriaceae* spp. abundances were not changing substantially, suggests the presence of functional redundancies across the larger fluctuating taxa. Many *Enterobacteriaceae* are facultative anaerobes that can use electron acceptors other than oxygen to carry out respiratory metabolism to drive ATP production. Given the genes that increase in abundance, this is a possible reason for their increased abundance. Heme production and sulfur assimilation are important for making iron-containing proteins which are important in the electron transport chain, ubiquinol and menaquinol are important electron carriers in bacterial electron transport chains and carnitine degradation can produce crotonobetaine which can serve as an anaerobic electron acceptor (Ghonimy et al., 2018; Kaila and Wikstrom, 2021). It may be interesting to test lower doses of this herb to determine whether the changes observed in Bacteroidetes and Proteobacteria are the result of inherent toxicity.

It is unknown which components of Hehuanhua promote the growth of *Enterobacteriaceae* and whether these are separate from the components that induce the beneficial effects of the herb. More research may help separate the two and generate a multi-compound drug with all the benefits and none of the negative side effects. In addition, more research is necessary to identify any microbially produced metabolites in the communities grown in the presence of these herbal extracts. It seems possible that Hehuanhua may induce significant host-bacterial crosstalk which deserves a more in-depth investigation but is outside the scope of the current study. This research has made it clear that along with other types of mechanistic studies, researchers should pursue gut microbiome interaction studies since the efficacy of some herbal remedies may indeed depend on their ability to modulate the microbiome.

## Data availability statement

The datasets presented in this study can be found in online repositories. The names of the repository/repositories and accession number(s) can be found below: https://www.ncbi.nlm.nih.gov/bioproject/PRJNA961974/.

## Ethics statement

The studies involving humans were approved by Ethics Committee of the University Hospital Ghent, Belgium (BC-09977). The studies were conducted in accordance with the local legislation and institutional requirements. The human samples used in this study were acquired from primarily isolated as part of your previous study for which ethical approval was obtained. Written informed consent for participation was not required from the participants or the participants’ legal guardians/next of kin in accordance with the national legislation and institutional requirements.

## Author contributions

JL: Conceptualization, Data curation, Formal Analysis, Methodology, Visualization, Writing – original draft, Writing – review & editing. AN: Conceptualization, Data curation, Formal Analysis, Validation, Visualization, Writing – review & editing. LL: Conceptualization, Project administration, Supervision, Writing – review & editing. JF: Conceptualization, Writing – review & editing. KM: Conceptualization, Writing – review & editing. PV: Conceptualization, Formal Analysis, Investigation, Methodology, Project administration, Supervision, Writing – review & editing. AB: Investigation, Methodology, Writing – review & editing. SD: Investigation, Methodology, Writing – review & editing. YL: Writing – review & editing, Investigation, Methodology. LY: Writing – review & editing, Supervision.

## Supplementary material

Supplementary Figure 1 Shows the relative contribution of different bacterial species to the genes within three unique pathways which are significantly upregulated with Hehuanhua treatment. These bacteria belong almost exclusively to the family *Enterobacteriaceae*.
