# Supplementary figures and images for: Impact of Baizhu, Daqingye, and Hehuanhua extracts on the human gut microbiome
Source: Front Cell Infect Microbiol. 2023 Dec 7;13:1298392. doi: 10.3389/fcimb.2023.1298392 (PMC10740150; doi:10.3389/fcimb.2023.1298392)

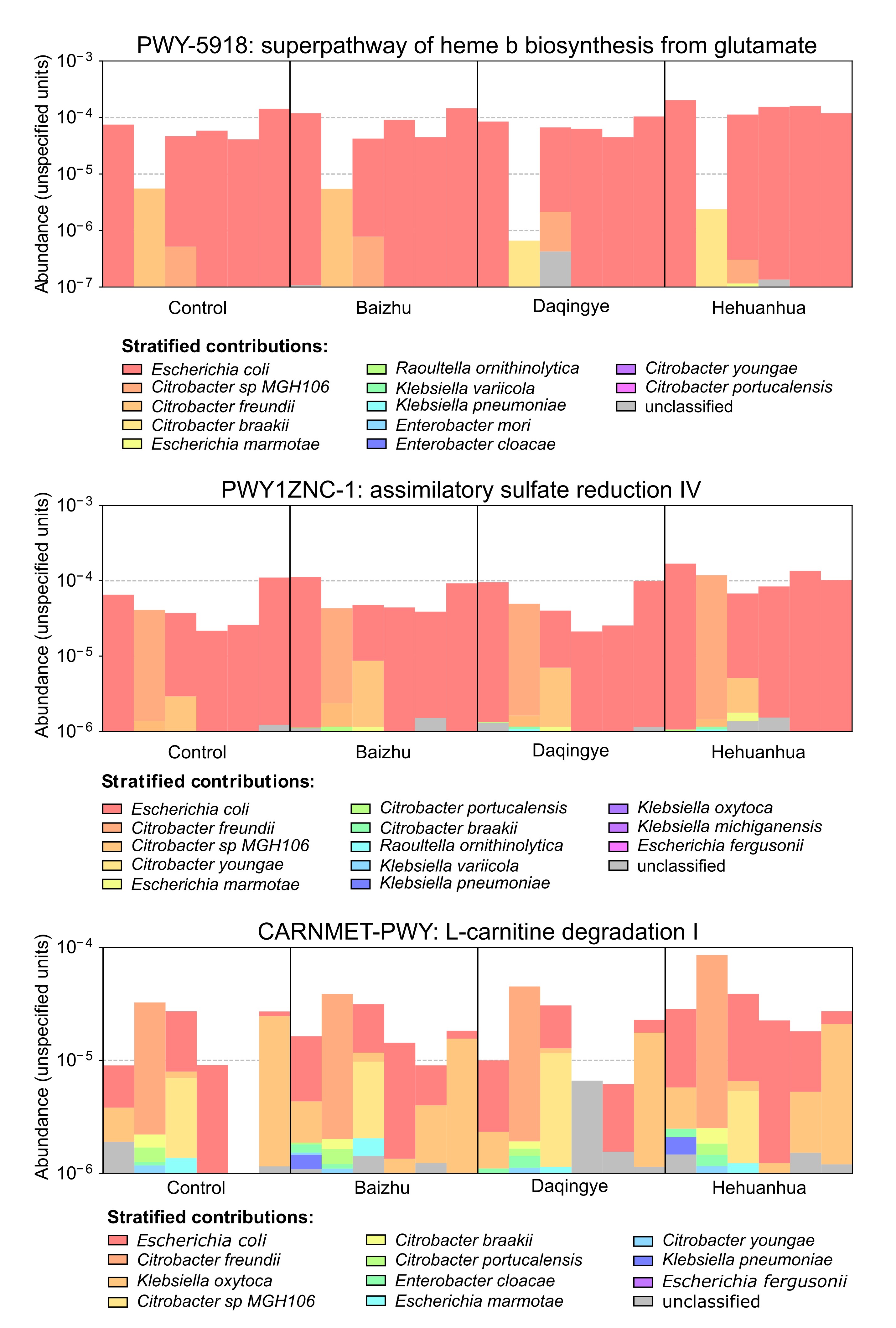

Supplement: Supplementary Figure 1 — Shows the relative contribution of different bacterial species to the genes within three unique pathways which are significantly upregulated with Hehuanhua treatment. These bacteria belong almost exclusively to the family Enterobacteriaceae. [file Image_1.jpeg]
